# Interleukin-1 mediates Alzheimer and Lewy body pathologies

**DOI:** 10.1186/1742-2094-3-5

**Published:** 2006-03-16

**Authors:** W Sue T Griffin, Ling Liu, Yuekui Li, Robert E Mrak, Steven W Barger

**Affiliations:** 1 Department of Geriatrics, University of Arkansas for Medical Sciences, Little Rock, Arkansas 72205, USA; 2 Department of Pathology, University of Arkansas for Medical Sciences, Little Rock, Arkansas 72205, USA; 3 Department of Neurobiology & Developmental Sciences, University of Arkansas for Medical Sciences, Little Rock, Arkansas 72205, USA; 4 Geriatric Research, Education and Clinical Center, Department of Veterans' Affairs Medical Center, Little Rock, Arkansas 72205, USA; 5 Mental Illness Research Education Center, Department of Veterans' Affairs Medical Center, Little Rock, Arkansas 72205, USA

## Abstract

**Background:**

Clinical and neuropathological overlap between Alzheimer's (AD) and Parkinson's disease (PD) is now well recognized. Such cases of concurrent AD and Lewy body disease (AD/LBD) show neuropathological changes that include Lewy bodies (α-synuclein aggregates), neuritic amyloid plaques, and neurofibrillary tangles (hyperphosphorylated tau aggregates). The co-occurrence of these clinical and neuropathological changes suggests shared pathogenic mechanisms in these diseases, previously assumed to be distinct. Glial activation, with overexpression of interleukin-1 (IL-1) and other proinflammatory cytokines, has been increasingly implicated in the pathogenesis of both AD and PD.

**Methods:**

Rat primary cultures of microglia and cortical neurons were cultured either separately or as mixed cultures. Microglia or cocultures were treated with a secreted fragment (sAPPα) of the β-amyloid precursor protein (βAPP). Neurons were treated with IL-1β or conditioned medium from sAPPα-activated microglia, with or without IL-1 receptor antagonist. Slow-release pellets containing either IL-1β or bovine serum albumin (control) were implanted in cortex of rats, and mRNA for various neuropathological markers was analyzed by RT-PCR. Many of the same markers were assessed in tissue sections from human cases of AD/LBD.

**Results:**

Activation of microglia with sAPPα resulted in a dose-dependent increase in secreted IL-1β. Cortical neurons treated with IL-1β showed a dose-dependent increase in sAPPα release, an effect that was enhanced in the presence of microglia. IL-1β also elevated the levels of α-synuclein, activated MAPK-p38, and phosphorylated tau; a concomitant decrease in levels of synaptophysin occurred. Delivery of IL-1β by slow-release pellets elevated mRNAs encoding α-synuclein, βAPP, tau, and MAPK-p38 compared to controls. Finally, human cases of AD/LBD showed colocalization of IL-1-expressing microglia with neurons that simultaneously overexpressed βAPP and contained both Lewy bodies and neurofibrillary tangles.

**Conclusion:**

Our findings suggest that IL-1 drives production of substrates necessary for formation of the major neuropathological changes characteristic of AD/LBD.

## Background

Parkinson's disease (PD), once thought of as a purely motor disorder, is linked to Alzheimer's disease (AD) in several ways. Many PD patients develop memory deficits, delirium, and other hallmarks of dementia late in the course of their disease [1,2]. Dementia with Lewy bodies is now a well-recognized entity featuring clinical cognitive impairment combined with the neuropathological finding of Lewy bodies in non-motor regions of the cerebral cortex. Such cortical Lewy bodies are often found in association with the amyloid plaques and neurofibrillary tangles pathognomonic for AD, and Lewy bodies correlate with clinical dementia in cases of mixed pathology [2]. Lewy bodies are composed of fibrillar aggregates of α-synuclein, and Trojanowski and colleagues [3] have shown that full length α-synuclein is present in Lewy bodies in cases of familial AD arising from βAPP mutation. From this, they propose that "the mechanisms of Lewy bodies formation are identical regardless of the biological trigger."

Our present study addresses potential roles for neuroinflammation and interleukin-1 (IL-1) in Lewy body formation. Pro-inflammatory cytokines such as IL-1, as well as other indicators of microglial activation, have been suggested as drivers of neuropathological changes in several neurodegenerative conditions. AD has been more extensively explored in this regard [5], but PD has also been associated with microglial activation [4]. In addition, other conditions that are associated with precocious development of AD-type neuropathological changes also show microglial activation and overexpression of IL-1 at early stages; these include Down's syndrome [6], AIDS-associated dementia [7,8], epilepsy [9,10], and traumatic brain injury [11].

It is increasingly clear that glial neuroinflammatory events are associated with neurodegenerative consequences. For instance, neuroinflammatory factors deleteriously impact normal regulation of neurotransmitters such as acetylcholine [12-14] and excitotoxins like glutamate [15] and D-serine [17], both of which could contribute to general declines in synapses [18] and specific declines in hippocampal NMDA-R1 receptors [16]. Moreover, each of these effects on neuronal function results in increases in the production of neuronal β-amyloid precursor protein (βAPP) and its secreted fragments (e.g., sAPPα) which, in turn, activate microglia and induce IL-1 synthesis and release [12]. These observations suggest that a chronic inflammatory process, driven mainly by activated microglia overexpressing IL-1, is primary in the development of many neurodegenerative conditions.

In this study, we used several experimental approaches to assess the effects of microglia-neuron interactions on neuronal production of βAPP, sAPP, α-synuclein, and phosphorylated tau. In addition, we demonstrated colocalization of activated microglia overexpressing IL-1 with neurons that overexpressing βAPP and simultaneously manifesting neurofibrillary tangles and Lewy bodies. Based on our results, we propose that neuronal-glial interactions give rise to co-stimulation of expression and release of sAPPα from neurons and excessive expression of IL-1 in activated microglia and that these interactions are key links in an array of self-propagating, molecular and cellular interactions that are neurodegenerative in nature, triggering the overlapping clinicopathological spectrums of AD, LBD, and PD.

## Methods

### Patients

Autopsy material was obtained from five patients with diagnoses of combined AD/LBD. In addition, one patient had a clinical diagnosis of PD. There were 2 males and 3 females, with an age range of 68–85 y. The patients whose tissue was examined postmortem, with appropriate consent, were participants in the University of Arkansas for Medical Sciences National Institute on Aging-sponsored Alzheimer's Disease Center under University Institutional Review Board guidelines.

### Reagents

Secreted APP was purified from a recombinant expression system as described previously [15]. Mouse IL-1ra was from R&D System (Minneapolis MN). Medium, serum, and B27 supplement for cell cultures were from Invitrogen (Grand Island NY).

### Cell cultures

Primary neuronal cultures were derived from the cerebral cortex of fetal Spraque-Dawley rats (embryonic day 18), as described previously [19]. Experiments using primary neuronal cells were performed after 8–12 days in culture. Primary cultures of rat microglia were generated from the cortical tissue of neonatal (0–3 days) Sprague-Dawley rats, as described previously [15]. When mixed cultures were used, primary microglia were added directly to cultures of primary neurons and exposed for 24 h to 0.0 to 10 ng/mL IL-1β. To test the potential role of factors derived from activated microglia in driving neuronal expression of substrates of AD- and PD-associated neuropathologies, cultures of primary neurons were incubated for 24 h with medium from unactivated primary microglia, or with medium derived from sAPP-activated (30 nM) microglia. To determine if such changes in expression were mediated by IL-1, some of the neuron cultures were treated with IL-1 receptor antagonist (IL-1ra, 50 ng/mL) before incubation with conditioned medium from activated microglia.

### Pellet implantation

Pellets impregnated with IL-1β (100 ng of recombinant mouse IL-1β) or control pellets (containing 100 ng of acetone-extracted bovine serum albumin, BSA) were implanted 2.8 mm caudal to bregma;4.5 mm right of the midline, and 2.5 mm deep to the pial surface [12]. Twenty-one male Sprague-Dawley rats weighing 264 ± 6 g were randomly divided into three groups. Eight rats received implants of IL-1-containing pellets, seven rats received pellets with BSA impregnation, and six rats served as un-operated controls. Twenty-one days after implantation, cortex from the hemisphere contralateral to the implant was collected for RNA isolation.

### RT-PCR

Total RNA was extracted from rat brain tissue with Tri-Reagent (Molecular Research, Cincinnati, OH). RT-PCR was performed as previously described [12]. The level of α-synuclein PCR product in each sample was normalized to that of GAPDH in the same sample.

### Western immunoblot assay

Western immunoblot analyses were performed as described previously [18]. Briefly, equal amounts of total protein (determined by bicinchonic acid assay) were resolved on 8–15% SDS/polyacrylamide gels and transferred to nitrocellulose blots. Blots were probed overnight at 4°C with either a rabbit anti-α-synuclein polyclonal antibody, diluted 1:1000 (Chemicon, Temecula CA); or a rabbit anti-βAPP polyclonal antibody, diluted 0.5 μg/mL (Stressgen, San Diego, CA; Vector Lab, Burlingame, CA); or monoclonal phosphorylated-Tau antibody, diluted 1:1000 (AT8, Pierce, Rockford, IL); or MAPK-p38, diluted 1:1000 (BioLabs, Inc., Beverly, MA); or monoclonal synaptophysin antibody, diluted 1:2000 (Roche, Indianapolis, IN); or IL-1β, diluted 0.1 μg/mL (Chemicon, Temecula, CA) After washing, blots were reacted with HRP-conjugated goat anti-rabbit antibodies, for polyclonal antibodies or anti-mouse for monoclonal antibodies. Detection of antibody-antigen binding was performed with Western-Light™ Chemiluminescent Detection System (Applied Biosystems, Foster City, CA). For Western immunoblot analysis of culture medium to detect either sAPP or IL-1β, media samples were concentrated via filtration through Centricon YM-10 centrifugal concentrators (Millipore, Bedford, MA) before analysis as above.

### Triple label immunohistochemistry

Analysis was performed on 6-micron thick tissue sections from formalin-fixed, paraffin-embedded blocks of parahippocampal gyri from brains of patients with AD/LBD. Tissue sections were deparaffinized by passing through a series of xylenes, and rehydrated through a graded series of alcohols, to deionized water. For the triple-labeled immunoreactions in the present study, the four antibodies were combined as follows: AT8 and α-synuclein with either IL-1α or βAPP. All incubations were followed by washing in PBS and, unless stated otherwise, were performed at room temperatures. For immunoreactions that included IL1α, AT8, and α-synuclein, sections were pretreated with an antigen retrieval enzyme digestion system (Digest-All 2, Zymed, South San Francisco, CA), immunoreacted at overnight with polyclonal IL-1α antibody (Peprotech, Rocky Hill, NJ), diluted 1:10 in 2.5% normal horse serum in PBS, immunoreacted with an ImmPRESS Reagent anti-rabbit Ig-peroxidase-micropolymerized reporter enzyme staining system (Vector), followed by detection with DAB (Zymed) and a 30-min treatment with Double Stain Enhancer (Zymed). Then the sections were pretreated for antigen epitope retrieval by microwaving in boiling 1 mM EDTA buffer (pH 8) for 10 min. Next, the sections were immunoreacted with AT8 antibody overnight, followed by a 30-min incubation with biotinylated goat-anti mouse antibody, diluted 1:200 in 2% normal goat serum in PBS, and then a 30-min treatment with avidin D-conjugated β-galactosidase (Vector), diluted 1:200 in 2% normal goat serum in PBS, and finally, detection using X-Gal Substrate Set (KPL). This antigen detection was followed by α-synuclein antigen epitope retrieval using two pretreatments: first, a 10-min microwave pretreatment in boiling 1 mM EDTA buffer (pH 8), and second, a 2-min incubation in concentrated formic acid, followed by immunoreaction with a monoclonal anti-α-synuclein antibody (Vector), diluted 1:30, for 2.5 h. This was followed by incubation with polymer-alkaline-phosphatase-conjugated secondary antibody (DAKO, Envision Kit), and labeled with permanent red (DAKO). When the three antigens to be detected were AT8, βAPP, and α-synuclein, monoclonal anti-βAPP antibody (Pierce) was immunoreacted in the place of polyclonal anti-IL-1α antibody in the protocol above as follows: sections were immunoreacted 30 min with a monoclonal AT8 antibody (Pierce), diluted 1:1000 in 2.5% normal horse serum in PBS, and immunoreacted with an ImmPRESS Reagent anti-mouse Ig-peroxidase-micropolymerized reporter enzyme staining system (Vector), followed by detection with DAB (Zymed), and then by a 30-min treatment with Double Stain Enhancer (Zymed). This was followed by α-synuclein antibody immunoreaction and detection by the β-galactosidase-avidin D/X-Gal system. Finally, anti-βAPP was applied and detected with the alkaline-phosphatase-conjugated secondary antibody and permanent red.

### Statistical analyses

The unpaired, two-tailed t-test was used to determine significance using the StatView 4.1 statistical analysis program.

## Results

### Mutual induction of IL-1β and sAPP in neuronal-microglial cultures

**Figure 1:**
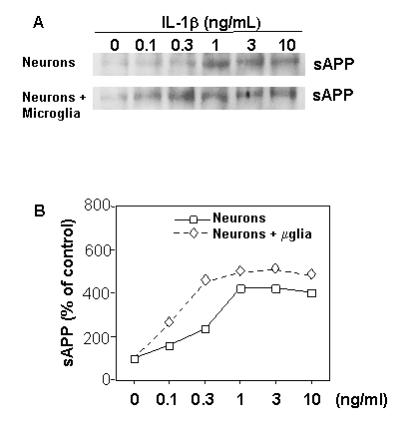
**Neuron-microglia interactions induce elevated expression of secreted APP (sAPP)**. Relative levels of sAPP were determined in medium from primary cortical neuron cultures treated with IL-1β (0.1–10 ng/mL) and in medium from IL-1β-treated neuron/microglia mixed cultures. Proteins in the medium were concentrated by Centricon, and sAPP was detected by western immunoblot analysis. **A**) Representative sAPP immunoblot; **B**) Densitometric quantification of immunoblot results. Values represent the mean ± SEM of three experiments (error bars are smaller than the datapoint symbols). At each IL-1β dose, the sAPP levels in medium of microglial neuronal-mixed cultures were greater than in medium from neurons cultured alone (*p *< 0.05).

To test specific degenerative feedback relationships between neurons and microglia, primary neuronal and microglial culture models were employed. IL-1β treatment of purified primary neurons, cultured alone, produced dose-dependent increases in sAPP release over an IL-1 concentration range of 0.1 and 1.0 ng/mL. These IL-1β effects were enhanced when the neurons were cultured together with microglia (Fig. 1). For instance, in such neuronal-microglial cultures, neuronal sAPP induction required as little as one third the amount of IL-1β required for a similar elevation of sAPP in the absence of microglia.

**Figure 2:**
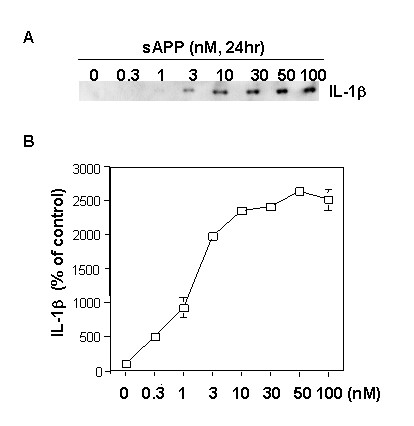
**Secreted APP (sAPP) induces dose-dependent increases in the levels of IL-1β released from primary microglial cultures**. Primary microglia were treated with the indicated doses of recombinant sAPPα for 24 h. Proteins in the medium were concentrated by Centricon filter and IL-1β was analyzed by western immunoblots. **A**) Representative IL-1β immunoblot; and **B**) Densitometric quantification of immunoblot data from at least three experiments.

One possible explanation for the enhanced overexpression of sAPP in mixed cultures, relative to that seen in neurons cultured alone, would be a positive feedback mechanism involving (i) IL-1β-induced sAPP release from neurons, (ii) sAPP-induced activation of microglia with induction of further IL-1β, and (iii) further induction of neuronal sAPP release by the increased levels of IL-1β. In support of this possibility, 24-h exposures of purified microglial cultures to recombinant sAPPα resulted in dose-dependent elevations of IL-1β expression (Fig. 2).

### The effects of conditioned media from sAPP-activated microglia on neuronal expression of α-synuclein, βAPP, phospho-MAPK-p38, and phospho-tau are mediated by IL-1

**Figure 3:**
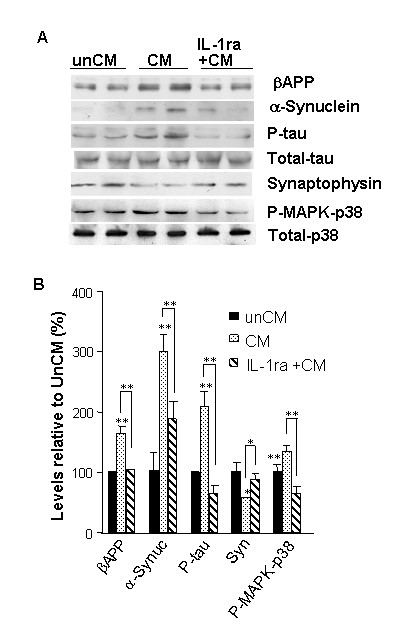
**IL-1 mediates microglia-induced elevation of neuronal levels of substrates of the neuropathological changes characteristic of AD and PD and Lewy body pathologies**. Primary neurons were cultured with the conditioned medium from either untreated primary microglia (**unCM**), or with medium from microglia that had been activated with 30 nM sAPPα (**CM**). Parallel neuron cultures were pretreated for 1 h with IL-1ra (50 ng/mL), followed by treatment with medium from sAPP-activated microglia (**IL-1ra + CM**). **A**) Representative western immunoblot using antibodies recognizing βAPP, α-synuclein, phosphorylated tau, synaptophysin, or p38-MAP kinase (MAPK-p38); **B**) Densitometric quantification of immunoblot results from duplicate experiments. * = p < 0.05, ** = p < 0.01.

To test the consequences of neuron-microglia interactions as they may apply to AD- and PD-related aspects of neurodegeneration, we assessed the role of IL-1 in the effects of conditioned medium from sAPP-activated microglia on neuronal cultures. Such exposure to sAPP resulted in an increase in the levels of α-synuclein that was mediated by IL-1, as demonstrated by suppression of α-synuclein expression by pretreatment of the neuron cultures with the natural IL-1 receptor antagonist IL-1ra (Fig. 3). α-Synuclein is a necessary component of the Lewy bodies that characteristize neuropathological features of both PD and dementia with Lewy bodies, and that often co-exist with the characteristic neuropathological features of AD. In addition to increased levels of α-synuclein, we found altered expression of other markers of neuropathological changes that have been found in AD, PD and AD/LBD. We found increases in the expression of *i*) βAPP – the precursor of Aβ and sAPP; *ii*) activated (phosphorylated) MAPK-p38, a tau kinase; and *iii*) phosphorylated tau, of the primary component of paired helical filaments and neurofibrillary tangles. Conversely, and coincident with these increases, we found *iv*) decreased levels of synaptophysin, a marker of synaptic integrity (Fig. 3). To test the role of IL-1 in these events, parallel cultures of neurons were pretreated for 1 h with IL-1ra (50 ng/mL) before application of the conditioned medium from sAPP-activated microglia. This pretreatment suppressed the increases in α-synuclein, βAPP, phosphorylated-tau, and activated MAPK-p38.

**Figure 4:**
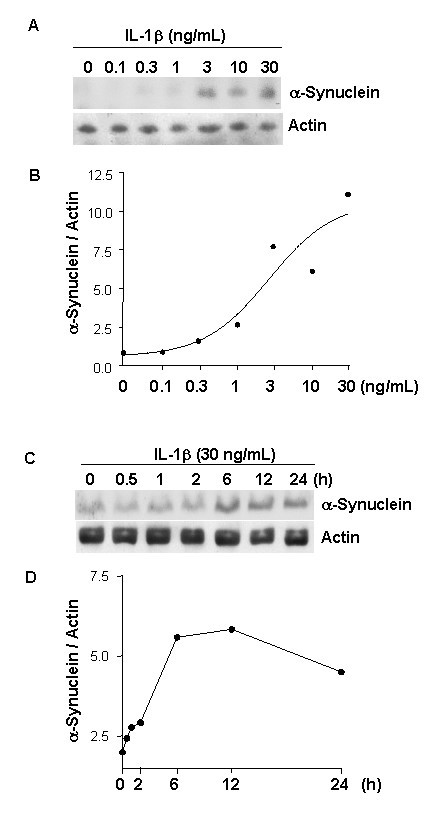
**IL-1 induction of α-synuclein is dose- and time-dependent**. Representative western α-synuclein (**α-synuc**) immunoblots of proteins from neurons treated with IL-1β for 24 h at the doses indicated (**A**) or with IL-1β at 30 ng/mL for the times indicated (**C**). **B**) and **D**) Densitometric quantification of immunoblot results from duplicate experiments.

To more carefully characterize the effect of IL-1β on α-synuclein expression, dependencies on dose and time were assessed in cultures of neurons. As little as 3.0 ng/mL IL-1β was an adequate dose to effect a substantial elevation of α-synuclein levels in neurons (Fig. 4A, B). Such elevations were apparent within 6 h of treatment (Fig. 4C, D).

### IL-1β induces α-synuclein *in vivo*

**Figure 5:**
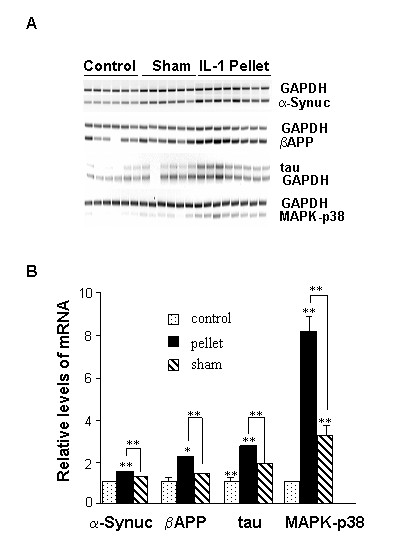
**Chronic exposure to excess IL-1β elevates expression of α-synuclein and other markers of AD and PD pathologies *in vivo***. Slow-release pellets containing IL-1β (**IL-1 pellet**) or BSA (**Sham**) were implanted into the cerebrum of adult Sprague-Dawley rats, and unoperated age-matched rats served as controls (**Control**). After 21 days in all cases, cerebral tissue was homogenized and RNA was isolated. RT-PCR was performed using primers specific for α-synuclein (**α-synuc**), βAPP, tau, or MAPK-p38. GAPDH was also analyzed as a quantitative control. **A**) RT-PCR results; **B**) Quantification of results by densitometric analyses. * = p < 0.05, ** = p < 0.01.

We next sought to extend our *in vitro *findings regarding IL-1-induced elevation of α-synuclein and of other proteins associated with neurodegenerative events in AD to an *in vivo *model. Pellets that slowly release IL-1β over a period of 21 days were implanted into the cerebral cortex of Sprague-Dawley rats; "sham" rats received pellets containing BSA [12]. IL-1β administration resulted in significant elevations in α-synuclein mRNA levels relative to either the levels observed in BSA "sham" animals (p < 0.02) or unoperated controls (p = 0.0003) (Fig. 5). Other markers of neurodegeneration elevated by IL-1β *in vitro *also showed elevated expression in cortices of rats bearing IL-1β pellets (Fig. 5).

**Figure 6:**
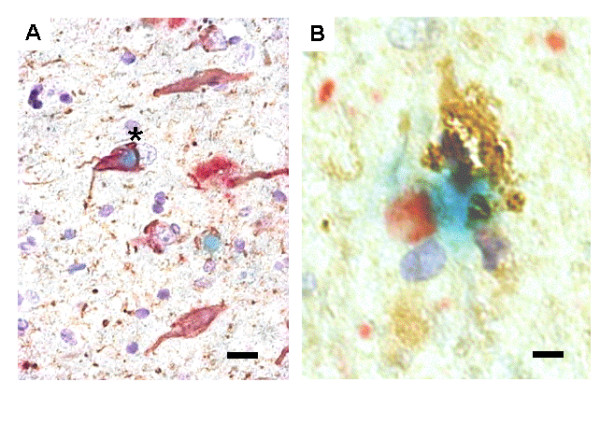
**Colocalization of activated microglia, overexpressing IL-1α, and markers of neuropathological changes in AD-LBD**. **A**) A neuron with a Lewy body (blue), neurofibrillary tangles (brown), and βAPP (red) colocalization. **B**) Localization of an activated microglia, overexpressing IL-1α (brown), with a neuron-like structure, containing a Lewy body (red) and neurofibrillary tangles (blue). Scale bar represents 15 μ (**A**) or 5 μ (**B**).

To determine whether the IL-1-mediated events observed *in vitro *and in brains of experimental animals are paralleled and mutually associated in AD/LBD, we examined histological sections from autopsy material. Figure 6 illustrates triple-label immunohistochemistry confirming colocalization of neurofibrillary tangles (anti-AT8) with Lewy body (anti-α-synuclein) staining (Fig. 6A), and the tell-tale accompaniment of microglial cell activation with over-expression of IL-1 (Fig. 6B).

## Discussion

We examined the effects of microglial activation on substrates of neuropathological elements of AD, PD, and AD/LBD using *in vitro *cell culture experiments and an *in vivo *pellet-implantation model. Activated microglia were found to elevate neuronal synthesis of βAPP and release of sAPP and increase levels of α-synuclein and phosphorylated-tau; microglial release of IL-1 appeared to be essential for each of these neuronal events. Moreover, a positive feedback mechanism was documented through which IL-1-induced sAPPα release stimulates further microglial IL-1β production. Chronic intracerebral delivery of IL-1β produced gene-expression changes consistent with the events observed in culture. Finally, we found that activated microglia overexpressing IL-1 colocalize with both AD- and PD-associated markers of neuropathology in human brain. These results, together with previous observations [6,20-22] suggest that microglia-derived IL-1 and neuron-derived sAPP are involved in a vicious circle of glia-neuronal interactions, which over time can precipitate neurodegenerative consequence common to both AD and PD.

The elevation of IL-1β levels by sAPPα and sAPPβ is a component of the microglial response to these proteins. In addition to this effect on IL-1β, nanomolar concentrations of both sAPPα and sAPPβ can activate microglia to express inducible nitric oxide synthase (iNOS), to release glutamate and D-serine, and to exhibit neurotoxicity; but sAPPβ lacks the balancing neuroprotective action of sAPPα [15,17,18,20]. As sAPPα and sAPPβ differ only at the carboxyterminus, their similar effects on microglial activation imply that a domain in the aminoterminus is responsible for such proinflammatory activity, confirmed by mutagenesis [15,20]. A receptor-binding event is further suggested by the fact that this same region of sAPP is required for binding by apolipoprotein E, an event that blocks microglial activation by sAPPs, possibly by stearic interference with sAPP binding events [20]. A receptor that mediates these effects has not been identified, but it may be related to that which mediates an activation of ERKs in PC12 cells [23]. This is especially compelling considering that sAPPα activates ERKs in microglia, where MAP kinases are necessary for microglial activation by sAPPα [24]. Interestingly, elevation of iNOS levels by sAPP appears to be mechanistically distinct from the elevation of iNOS by Aβ or LPS [25].

We have previously shown that neuronal stress increases neuronal synthesis of βAPP and secretion of sAPP, which in turn activates microglia and increases IL-1 synthesis [20] and release [12]. IL-1 is *sufficient *to elevate expression and processing of βAPP, which could favor production of either amyloid β-peptide or sAPPα [26]. The mechanisms involved in the elevation of βAPP processing appear to be complex. IL-1 can apparently elevate the expression of βAPP by transcriptional [27] and post-transcriptional events [28]. Once expressed, βAPP can be dramatically altered in its processing by IL-1 [29,30]. The cytokine has been shown to elevate processing by both γ-secretase [31] and α-secretase [32]. The latter, relevant to the stimulation of sAPP levels by IL-1 reported here, appears to require MAP kinases of the MKK1 and JNK classes, at least in neuroglioma cells [32].

In addition to its sufficiency for βAPP expression and processing, we show here that IL-1 is also *necessary *for the pathogenic signaling that activated microglia exert on neurons leading to diverse pathological changes, including elevation of α-synuclein levels. IL-1 also induces tau phosphorylation and depression of synaptophysin levels [18], actions that are consistent with AD pathology. Thus, an inflammatory cytokine can lead to the convergence of neuropathological events that are traditionally considered AD-associated (e.g., NFTs) with some that are not (e.g., Lewy bodies). The particular constellation of neuropathological markers occurring in association with one another in a given individual or a given neuron may depend upon coincidences of genetics, neurochemical profile, or various other autonomous variables rather than any differential the exposure to IL-1.

Our results suggest that the contributions of IL-1 to AD pathology include influences of this cytokine on synaptic function [33]; namely, (*i*) a decrease in synaptophysin, perhaps indicative of inflammation-related synapse loss, and (*ii*) an increase in α-synuclein, perhaps in an attempt to repair this very event [34]. The effect of IL-1β on α-synuclein in cultures of primary neurons is generally consistent with prior reports that the cytokine induces α-synuclein protein in macrophages [35] and a glioma cell line but not in primary astrocytes [36]. Negative data were also acquired in a teratocarcinoma cell line, albeit one that is similar to neurons when differentiated [37]. These findings do not refute the data we obtained with post-mitotic, primary neurons. α-Synuclein is normally found as an abundant synaptic vesicle protein of unclear function. And while it can be found in glia under both normal [38] and pathological conditions [39], its accumulation is much more prominent as Lewy bodies in neurons in PD and AD/LBD [40,41]. Mice transgenically modified to overproduce human forms of both Aβ and α-synuclein have higher rates of α-synuclein aggregation [42], a phenomenon that now appears to have been confirmed in humans with Lewy body disease [43].

## Conclusion

Our findings regarding the involvement of IL-1 in microglia activation-induced neuronal overexpression of βAPP and α-synuclein provide a mechanistic link between neuronal stress, microglial activation, IL-1 overexpression, and sAPP-driven events, leading to the recognized neuropathological changes that encompass both AD and PD. These interrelated events appear to be triggered in conditions that increase risk for precocious development of AD, as well [22,44]. Based on the quantitative studies reported here, particularly the observations in rats implanted with IL-1-containing pellets, one could predict that any neural condition involving IL-1 overexpression would exhibit concomitant pernicious alterations in the substrates associated with the hallmark neuropathologies in either AD or PD, alone or together. IL-1-mediated downstream consequences may favor manifestations or precocious development of AD, PD, or AD/LBD, a distinction likely dependent on the originating insult or the specific neuronal cell type first affected. For instance, in Down's syndrome and familial AD, genetic forces are the most likely culprits; in head injury and epilepsy, direct neuronal trauma; in AIDS, viral infection of microglia; and in PD and sporadic AD, unknown insults directed in a cell-specific manner to the affected neurons. As we show here, excessive levels of IL-1 can alter the expression of the neuronal substrates of AD, PD, and AD/LBD, thus predisposing the brain for establishment of a cycle of neuronal compromise, consequential activation of glia, and further IL-1 overexpression. Therefore, because of the potential of IL-1 to drive alterations in neuronal expression of disease substrates, we propose overexpression of CNS IL-1 as a drug discovery target for therapeutic intervention in IL-1-mediated self-propagating cycles.

## Competing interests

The author(s) declare that they have no competing interests.

## Authors' contributions

This study is based on an original idea of WSTG, who directed the work with SWB. SWB, LL, and WSTG wrote the manuscript. REM provided expertise on Lewy body disease, provided neuropathological evaluation of tissues, and participated in preparation of the final manuscript. SWB produced the recombinant sAPP. LL, in collaboration with YL, performed the experiments, constructed the figures, and made important conceptual contributions.
